# Emerging pathogens in urinary tract infections: virulence and phenotypic characterization of *Pseudomonas aeruginosa* strains

**DOI:** 10.1128/msphere.00151-26

**Published:** 2026-05-13

**Authors:** Rachel C. Fleck, Olena Gorodyna, Hao Zhou, Hannah Bryant, Mihaela Gadjeva, Allyson E. Shea

**Affiliations:** 1 Department of Microbiology and Immunology, College of Medicine, University of South Alabama 5557, Mobile, Alabama, USA; 2 Department of Biological Sciences, College of Medicine and Health Sciences, Abu Dhabi, United Arab Emirates; 3 Center for Biotechnology (BTC), Khalifa University, Abu Dhabi, United Arab Emirates; 4 Department of Infectious Disease Research, Moderna TX 692728 , Cambridge, Massachusetts, USA; The University of Iowa, Iowa City, Iowa, USA

**Keywords:** urinary tract infection, *Pseudomonas aeruginosa*, uropathogen, virulence factors, pathogenesis

## Abstract

Urinary tract infections (UTIs) affect a broad patient population and inflict a substantial financial burden on the U.S. healthcare system. While uropathogenic *Escherichia coli* (UPEC) causes the majority of cases, other pathogens are emerging. Patient data from our healthcare system in the Gulf Coast region of Alabama revealed that *Pseudomonas aeruginosa* accounted for 4.0% of UTI cases, roughly double the national average, prompting further investigation into this historically understudied uropathogen. Here, we performed whole-genome sequencing and phenotypic assays on 55 urinary *P. aeruginosa* isolates to characterize genomic diversity, antibiotic resistance, and virulence-associated phenotypes in the context of UTI. Multilocus sequence typing identified 19 strains with novel sequence types, underscoring the uncharacterized diversity of urinary *P. aeruginosa* isolates. Serotype O6 was most common and enriched in patients with indwelling catheters, whereas O4 was linked to diabetes mellitus. Antibiotic susceptibility testing (AST) revealed high levofloxacin resistance (29.1%), with 14.5% multidrug-resistant (MDR) and 9.1% extensively drug-resistant (XDR) isolates. Resistance patterns correlated with demographics, including higher meropenem and aztreonam resistance among isolates from African American patients. Phenotypic assays of growth, motility, and biofilm formation revealed negative correlations between antibiotic resistance and virulence. Specific virulence genes correlated with enhanced iron acquisition, hemolysis, and *in vivo* colonization. Notably, motility and exotoxin profiles were associated with *P. aeruginosa* ascension in a murine UTI model. Together, these findings provide insight into the diversity, resistance, and virulence characteristics of urinary *P. aeruginosa* isolates and highlight the need for further study of this emerging uropathogen.

**IMPORTANCE:**

*Pseudomonas aeruginosa* is an emerging but understudied pathogen in urinary tract infections (UTIs). Given its resilience, adaptability, and the growing threat of multidrug resistance, *P. aeruginosa* remains a significant challenge in clinical microbiology and infection control. Using genomic sequencing, phenotypic assays, a murine infection model, and detailed patient metadata, we characterized 55 urinary clinical isolates and identified substantial genomic diversity, including 19 strains with previously undescribed sequence types. We observed associations between antibiotic resistance patterns and patient demographics, as well as a negative relationship between resistance and virulence phenotypes. In addition, variations in traits such as flagellar alleles and exotoxin profiles were associated with differences in urinary tract colonization. These findings highlight the diversity of urinary *P. aeruginosa* isolates and identify bacterial traits that may contribute to infection in the urinary tract.

## INTRODUCTION

*Pseudomonas aeruginosa* is a Gram-negative, opportunistic pathogen best known for causing severe lung and wound infections, yet it can also pose significant challenges in the urinary tract (1). Although urinary tract infections (UTIs) are predominantly caused by uropathogenic *Escherichia coli* (UPEC), *P. aeruginosa* accounts for up to 10% of catheter-associated urinary tract infections (CAUTIs) and 16% of UTIs in intensive care units (ICUs) (2–4). However, in contrast to extensive studies in lung and wound infection models, the characteristics that allow *P. aeruginosa* to successfully colonize the urinary tract remain poorly understood.

*P. aeruginosa* persists in diverse environments through a combination of intrinsic and acquired antibiotic resistance mechanisms and a wide array of virulence traits (4, 5). Clinical isolates are often multidrug-resistant (MDR) or extensively drug-resistant (XDR), making treatment especially challenging (5–7) and contributing to the CDC designation of MDR *P. aeruginosa* as a serious public health threat (8). Biofilm formation, particularly on urinary catheters, further enhances resistance by protecting bacteria from host defenses and antibiotic penetration (1). Biofilm development is closely tied to motility and surface attachment mechanisms involving flagellar genes (type A *flaA* and type B *fliC*) and pili genes such as *pilA* (1, 9). These processes are further coordinated by quorum sensing (QS), which controls biofilm formation and virulence expression through key transcriptional regulators such as *lasR* and *rhlR* (10). Iron acquisition is another important determinant of *P. aeruginosa* virulence. The iron-scavenging siderophore pyoverdine, partially encoded by *pvdA*, supports growth in iron-limited environments such as the urinary tract (11–13). Given the rise of antibiotic resistance, these virulence mechanisms have emerged as targets of anti-virulence therapies (14, 15). Indeed, experimental disruption of QS pathways has been shown to significantly reduce virulence in the urinary tract (16, 17), highlighting the importance of researching these mechanisms to better understand and treat *P. aeruginosa* UTIs.

Another critical virulence feature is the secretion of exotoxins, particularly via the type III secretion system (T3SS). The T3SS injects cytotoxins such as ExoS, ExoT, ExoU, and ExoY directly into host cells, disrupting cytoskeletal integrity, interfering with immune signaling, and promoting cell death (18–21). Among these, ExoU and ExoS are the most clinically relevant and are generally considered mutually exclusive among strains (22, 23). ExoU is strongly associated with virulence, severe cytotoxicity, and poor clinical outcomes, particularly in the context of pneumonia (24, 25).

Variation in *P. aeruginosa* O-antigen serotypes can also influence infection severity and treatment outcomes. For example, O6 and O11 have been associated with increased mortality in patients with pneumonia (26), and O11 isolates often harbor *exoU* and multidrug resistance (27, 28). Given their pathogenic potential, O-antigens have recently been investigated as vaccine and therapeutic targets, underscoring the importance of characterizing O-antigen serotypes in clinical *P. aeruginosa* isolates (29). Several patient factors are also known to increase the risk of *P. aeruginosa* UTIs, including indwelling urinary catheters (IDCs), immunosuppressive therapies, diabetes mellitus (DM), and prolonged hospitalization (7). However, the roles of other host factors in *P. aeruginosa* infection and virulence in the urinary tract remain relatively understudied, highlighting the need for integrative studies that investigate both pathogen diversity and patient characteristics in the context of UTI.

Here, we phenotypically and genotypically assessed 55 *P. aeruginosa* clinical urinary isolates from a diverse patient population. We performed bacterial growth, biofilm formation, iron acquisition, cytotoxicity, and motility assays to directly assess *P. aeruginosa* virulence and persistence *in vitro*. We also conducted whole-genome sequencing (WGS) to identify virulence and antibiotic resistance genes as well as characterize the multi-locus sequence type (MLST) and O-antigen serotype of each strain. Integrating these results with abundant patient metadata, we examined relationships between bacterial genotype, phenotype, and host variables to identify factors associated with *P. aeruginosa* pathogenicity in UTIs.

## RESULTS

### Urinary *P. aeruginosa* isolates exhibit substantial genomic diversity

*P. aeruginosa* accounts for only 1% of uncomplicated UTIs and 2% of complicated UTIs nationally (3); however, we report a prevalence of 4.03% in our local healthcare system (Fig. S1). This discrepancy prompted us to investigate the genotypic and phenotypic traits of circulating urinary isolates. Fifty-five *P. aeruginosa* clinical isolates were obtained from urine cultures at the University of South Alabama, representing a diverse cohort with various comorbidities (Table 1). Three reference strains were included in this study for comparison: PAO1, PA103, and ATCC 27853, isolated from wound, lung, and bloodstream infections, respectively (30–33). Indeed, there is no *P. aeruginosa* urinary type strain for comparison.

**TABLE 1:** Patient demographics and comorbidities

Characteristic	Value
Age (years)	
Median (range)	71 (1–92)
Sex	
Male	26 (47.3%)
Female	29 (52.7%)
Race	
African American	18 (32.7%)
White	37 (67.3%)
Ethnicity	
Hispanic	2 (3.6%)
Not Hispanic	53 (96.4%)
BMI	
Median (range)	27.3 (17–65)
Missing	9 (16.4%)
Co-Infection	
Yes	5 (9.1%)
No	50 (90.9%)
Comorbidities	
Sepsis	7 (12.7%)
Diabetes mellitus (DM)	22 (40.0%)
Recurrent UTI (rUTI)	41 (74.5%)
Indwelling catheter (IDC)	23 (41.8%)

**Fig 1:**
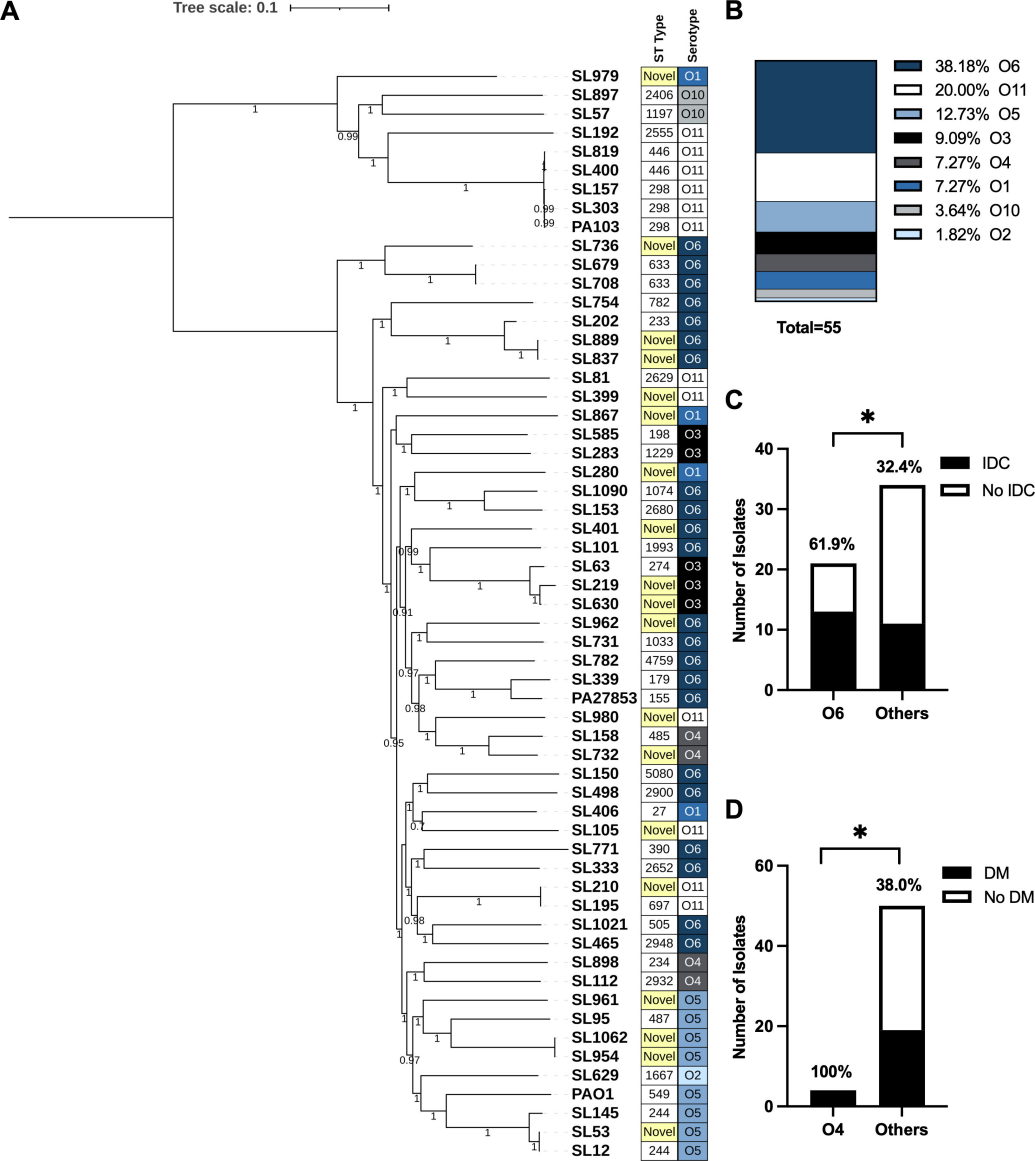
Genotypic and phylogenetic characterization of 55 *Pseudomonas aeruginosa* UTI clinical isolates. (**A**) Phylogenetic tree of 55 *P. aeruginosa* clinical isolates and type strains PAO1, PA103, and ATCC 27853 with ST types (novel highlighted in yellow) and serotypes indicated. Bootstrap values >0.7 are displayed. Tree is rooted at the midpoint. (**B**) Serotype prevalence in the clinical isolates (*n* = 55) determined by PAst. Bar graphs indicate the number of isolates from (**C**) patients with indwelling catheters (IDCs) for serotype O6 vs all other serotypes and (**D**) patients with diabetes mellitus (DM) for serotype O4 vs all others. Fisher’s exact test was used to determine significance between groups (*P < 0.05).

Whole-genome sequencing (WGS) followed by SNP-based phylogenetic analysis revealed substantial genomic diversity among clinical isolates (Fig. 1A). Multilocus sequence typing (MLST) assigned 36 isolates to 34 unique sequence types (STs), while the remaining 19 isolates did not match existing PubMLST profiles and were designated as novel (Data set S1). Although most isolates represented unique STs, four lineages (ST446, ST298, ST633, and ST244) were detected in multiple isolates. As expected, isolates sharing the same ST clustered closely together on the phylogenetic tree (34, 35). Comparison with publicly available U.S. urinary *P. aeruginosa* isolates revealed notable differences in ST distribution (Fig. S2A). The globally disseminated high-risk lineage ST235 comprised 28.2% of national urinary isolates but was absent in our cohort (Fig. S2B). Conversely, 32.7% of isolates in our cohort lacked previously assigned ST classifications compared to 0% among the national urinary isolates (Fig. S2C).

O-antigen serotyping identified eight distinct serotypes in our cohort, with O6 being the most prevalent (38.18%) (Fig. 1B). Unlike STs, isolates sharing the same serotype were distributed across multiple phylogenetic clades (Fig. 1A). When analyzed in the context of patient variables, the O6 serotype was enriched in patients with indwelling catheters (IDCs) (Fig. 1C) and remained independently associated with IDC status after multivariable adjustment (OR 3.85, 95% CI 1.19–13.60; Table S1). Similarly, all O4 isolates (4/4) were detected in patients with diabetes mellitus (DM) (Fig. 1D). These findings demonstrate substantial genotypic diversity among urinary *P. aeruginosa* isolates in our cohort and motivate further investigation into how this genetic variation shapes antibiotic resistance and virulence phenotypes.

### Patient demographics associate with specific antibiotic resistance profiles

**Fig 2:**
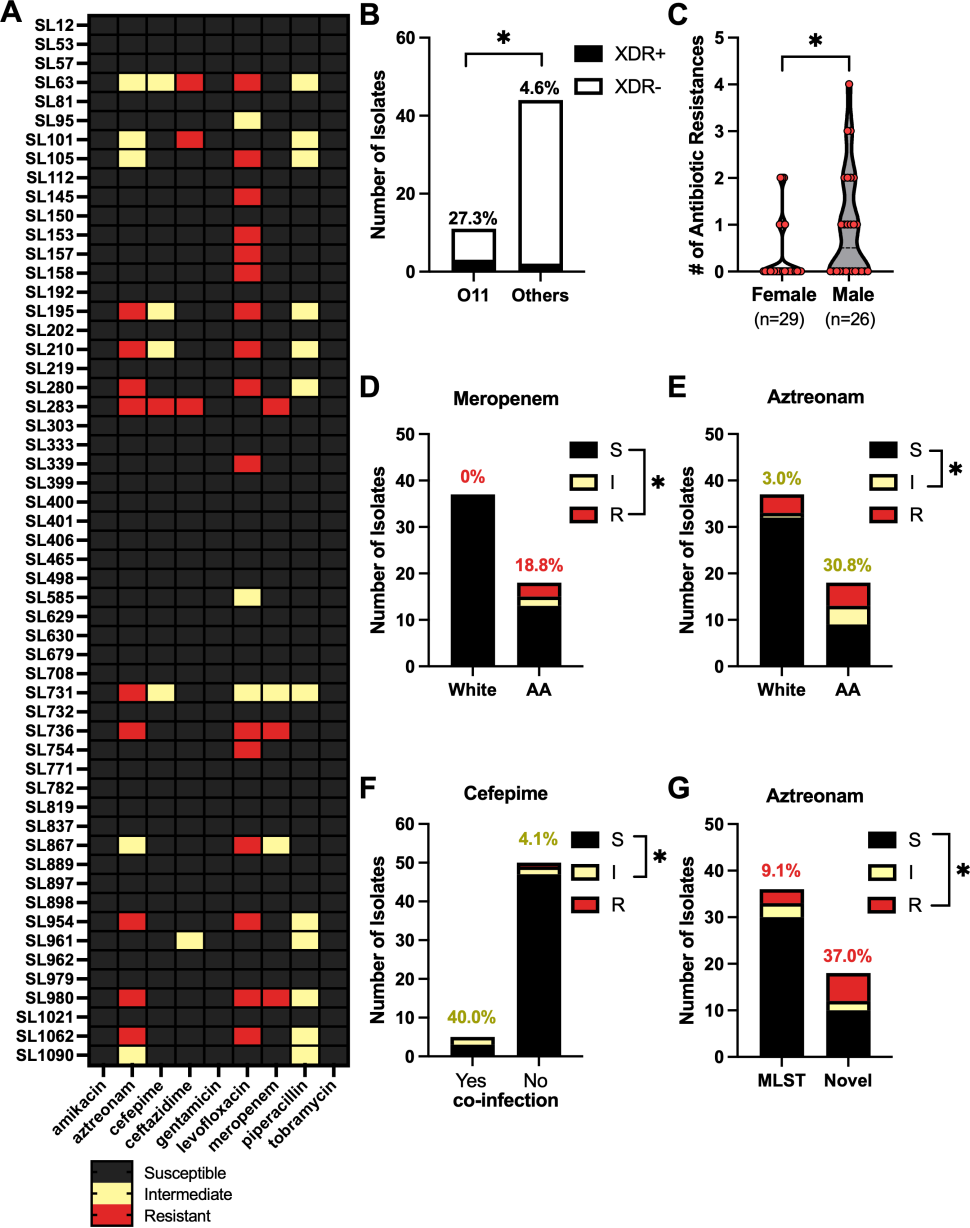
Antimicrobial resistance in *P. aeruginosa* isolates is associated with patient race, sex, and strain type. (**A**) Antibiotic susceptibility testing (AST) was performed, and strains were classified as susceptible (S; shown in black), intermediate (I; shown in yellow), or resistant (R; shown in red) to antibiotics based on the minimum inhibitory concentrations (MICs). (**B**) Prevalence of extensive drug-resistance (XDR, shown in black) in strains with the O11 serotype vs all other serotypes. (**C**) Violin plot of the total number of antibiotic resistances (R or I) per strain in isolates from females and males. Each point represents a clinical isolate. (**D and E**) Stacked bar graphs representing the median number of isolates from White and African American (AA) patients with (**D**) meropenem resistance and (**E**) aztreonam intermediate resistance. (**F**) Stacked bar graph of cefepime intermediate susceptibility in isolates from polymicrobial vs monomicrobial UTIs. (**G**) Stacked bar graph representing aztreonam susceptibility in isolates with established ST types (MLST) vs novel ST types (Novel). Fisher’s exact test was used to determine significance of all panels except panel C, in which Mann-Whitney *U* test was used (*P < 0.05).

Antimicrobial susceptibility testing (AST) revealed a wide range of resistance profiles (Fig. 2A). Levofloxacin resistance was the most common, with 29.1% (16/55) of isolates conferring resistance. Of the 55 strains, 8 (14.5%) were multidrug-resistant (MDR) and 5 (9.1%) were extensively drug-resistant (XDR) (Data set S2). Isolates of the O11 serotype were 7.9 times more likely to be XDR (Fig. 2B) and remained significantly associated with XDR isolates after adjustment for IDC status, a recognized risk factor for multidrug-resistant UTI (7, 36) (OR 7.74, 95% CI 1.10–68.00; Table S1).

Interestingly, resistance patterns varied by specific patient demographics and comorbidities. Isolates from male patients harbored significantly more resistances per strain than those from female patients (Fig. 2C), with 50% of male-derived isolates and 24% of female-derived isolates having one or more resistance. Isolates from African American patients were significantly more likely to be resistant to meropenem (Fig. 2D) and 14.2 times more likely to have intermediate aztreonam resistance (Fig. 2E) compared to those from White patients. Intermediate cefepime resistance was more common among strains isolated from polymicrobial infections than single causative agent cases (Fig. 2F). Aztreonam resistance was enriched among isolates designated as novel sequence types (Fig. 2G) and remained associated after adjustment for race (OR 4.44, 95% CI 0.97–24.21; Table S1). However, given the limited number of resistant isolates (*n* = 9) and wide confidence intervals, this finding should be interpreted cautiously.

To complement phenotypic testing, we assessed antibiotic resistance genotypically using the Comprehensive Antibiotic Resistance Database (CARD) Resistance Gene Identifier (RGI) (37) and identified numerous intrinsic resistance loci across the cohort (Fig. S3). Because antimicrobial resistance in *P. aeruginosa* often arises through chromosomal mutations, we next examined quinolone resistance-determining regions (QRDRs) for mutations known to confer fluoroquinolone resistance (38–40). This analysis revealed GyrA mutations in 8 of the 17 levofloxacin-resistant isolates, most commonly the T83I substitution accounting for approximately 47% of fluoroquinolone resistance in this cohort (Data set S3). Together, these findings demonstrate that resistance phenotypes reflect both intrinsic resistance determinants and additional chromosomal mechanisms not captured by gene presence alone.

### Strain background and polymicrobial infection status correlate with differential growth in human urine

**Fig 3:**
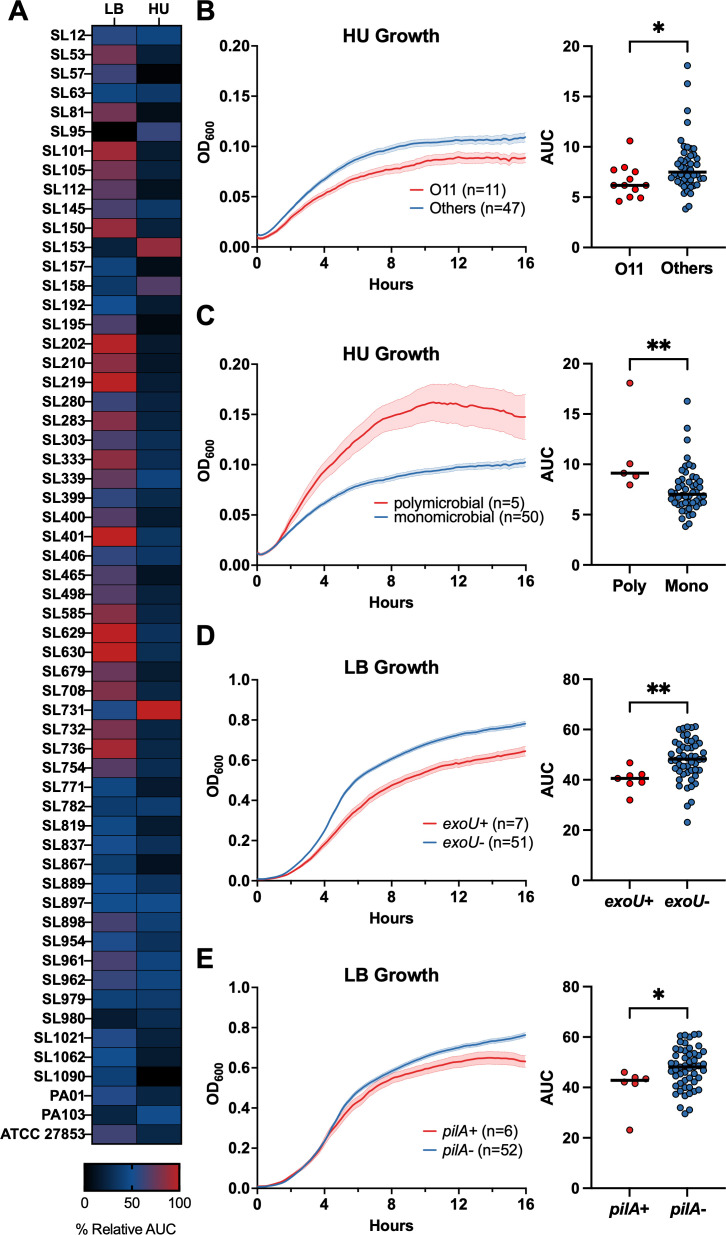
Virulence genes and polymicrobial infection are associated with growth of *P. aeruginosa*. (**A**) Growth of *P. aeruginosa* clinical isolates and type strains PAO1, PA103, and ATCC 27853 in LB broth (LB) and filter-sterilized pooled human urine (HU) displayed in a heatmap of normalized area under the curve (AUC) (*n* = 3–8). Data were normalized to the smallest (0%) and largest (100%) values in the data set. (**B–E**) Growth curves are displayed as OD_600_ over time. The data are an average of all strains with ± SEM shaded. The dot plot shows the average AUC for each strain with a solid black line to indicate median. (**C**) Growth in human urine of clinical isolates from polymicrobial (red) vs monomicrobial (blue) UTIs. Growth in LB of clinical isolates (**D**) with *exoU* (red) vs without *exoU* (blue) and (**E**) with PAO1-like PilA homologs (red) vs without (blue). Statistical significance was determined via Mann-Whitney *U* test (*P < 0.05, **P < 0.01).

Many clinical isolates outgrew the reference strains in either LB or human urine, but none excelled in both, suggesting a metabolic trade-off between growth in nutrient-rich and nutrient-limited environments as observed in other species such as uropathogenic *E. coli* (41, 42) (Fig. 3A). Growth in human urine was enhanced by 31.9% in strains with the O5 serotype (Fig. S4A), while strains with serotype O11 exhibited a 28.9% growth decrease (Fig. 3B) compared to other serotypes. Strains isolated from polymicrobial UTIs showed a 36.6% increase in human urine growth (Fig. 3C) compared to isolates from single organism infections. Isolates with BLAST-detected PAO1 ParS homologs also exhibited a 31.9% increase in growth (Fig. S4B). After adjustment, only polymicrobial infection status and O11 serotype remained associated with growth in human urine (Table S4). In LB, *exoU*-positive strains (Fig. 3D) and strains with PAO1-like PilA homologs (Fig. 3E) displayed significantly reduced growth. Interestingly, no patient-associated variables aside from co-infection correlated with pathogen growth (Fig. S5). Overall, these findings suggest that differences in genomic background contribute to variation in growth across distinct nutrient conditions.

### Iron acquisition and hemolytic activity are associated with virulence loci

**Fig 4:**
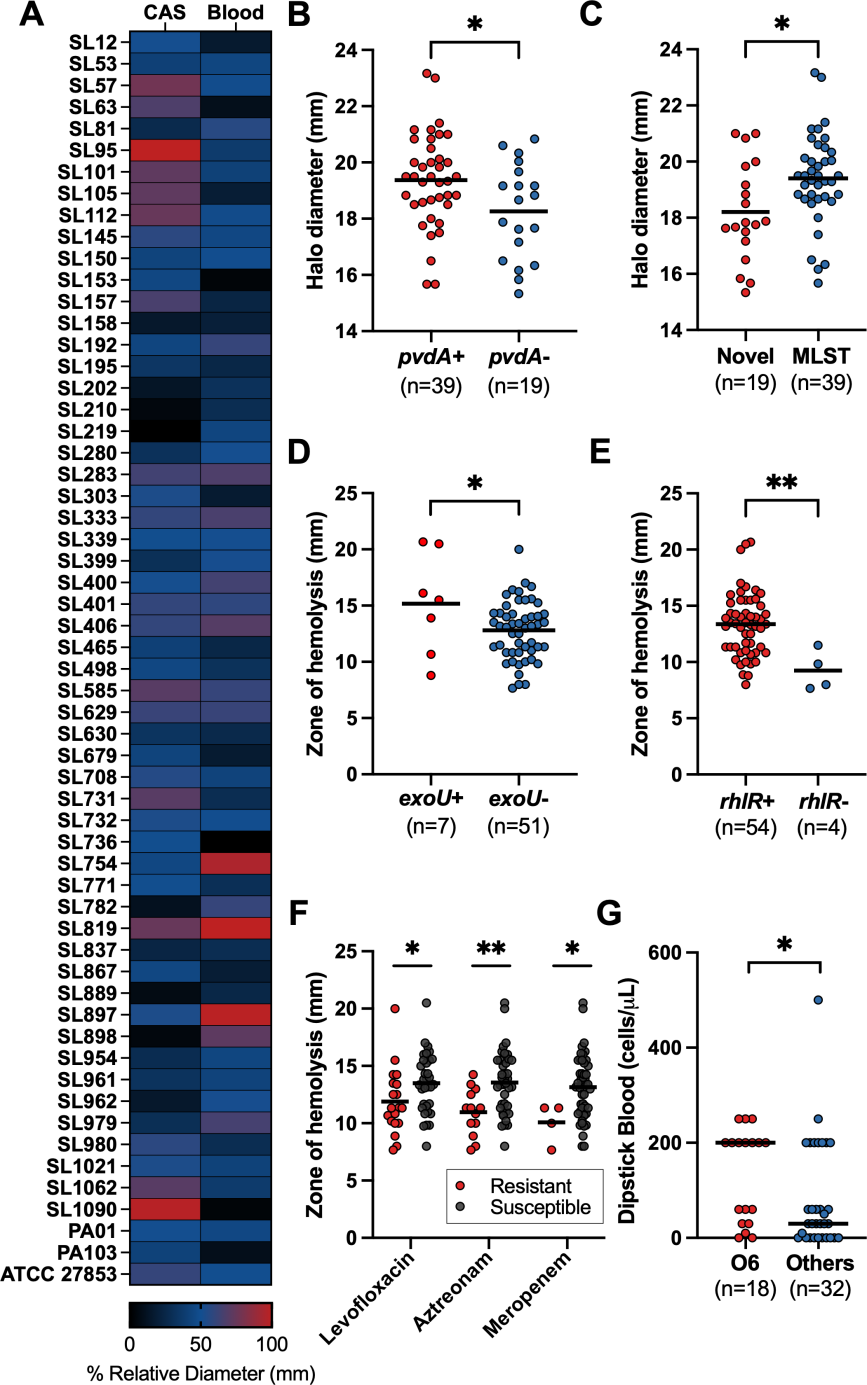
Siderophore production and hemolytic activity vary with toxin-encoding genes and antibiotic resistance. *P. aeruginosa* clinical isolates and type strains were tested on chrome azurol S (CAS) plates to assess siderophore production and blood agar plates to assess hemolytic ability. (**A**) Heatmap of normalized CAS and blood agar plate zones of inhibition (mm). Data were normalized to the smallest (0%) and largest (100%) values in each data set. CAS halo diameter (mm) by (**B**) PvdA sequence similarity to the PAO1 reference and by (**C**) ST type novelty. Each dot represents the average of biological replicates for an individual strain, and black lines indicate the mean of all strains in a group. Hemolytic zone of clearance (mm) in strains with and without (**D**) *exoU* and (**E**) a PAO1-like RhlR homolog. (**F**) Zone of hemolysis (mm) by resistance to levofloxacin, aztreonam, and meropenem. Resistant strains are shown as red dots, and susceptible strains are black dots. Mean is indicated as a solid line. Statistical significance was determined via unpaired *t*-test for panels **B–F** (*P < 0.05, **P < 0.01). Welch’s *t*-test was performed as a sensitivity analysis and resulted in the following *P* values for each panel: B = 0.0244, C = 0.0178, D = 0.2196, E = 0.0118. (**G**) Urinalysis results from USA Hospital were obtained for the UTI urine samples of origin for the 55 clinical isolates. A plot is shown of blood cells in urine specimens containing *P. aeruginosa* strains of serotype O6 versus other serotypes. Statistical significance was determined via Mann-Whitney *U* test (*P < 0.05) for panel **G**.

Iron acquisition and cytotoxicity are critical determinants of *P. aeruginosa* pathogenicity, influencing both survival in the iron-limited urinary tract and the extent of host tissue damage (1, 13). Most strains demonstrated either strong iron acquisition or hemolytic activity, but strain SL819 was an exception that displayed increased activity in both assays (Fig. 4A). Siderophore production was modestly higher (6.1%) in strains with PAO1-like PvdA homologs (Fig. 4B). In contrast, isolates with novel sequence types (Fig. 4C) showed a 6.2% reduction in iron chelation compared to known MLST strains. Hemolytic activity was 18.4% higher in *exoU*-positive isolates (Fig. 4D) and 44.5% higher in isolates with BLAST-detected RhlR homologs (Fig. 4E), consistent with the known roles of these genes in blood cell lysis (43, 44). In contrast, strains resistant to levofloxacin, aztreonam, or meropenem demonstrated reduced hemolytic activity (Fig. 4F), suggesting a potential trade-off between cytotoxicity and antibiotic resistance. Furthermore, O6 serotype strains were associated with 6.6-fold higher blood urine concentrations than other serotypes (Fig. 4G). Collectively, these findings highlight that serotype and toxin-related loci are associated with host damage and may also influence the urinary niche, while certain resistance traits are associated with reduced cytotoxic ability.

### Motility and biofilm phenotypes vary across strain types, virulence loci, and flagellar alleles

**Fig 5:**
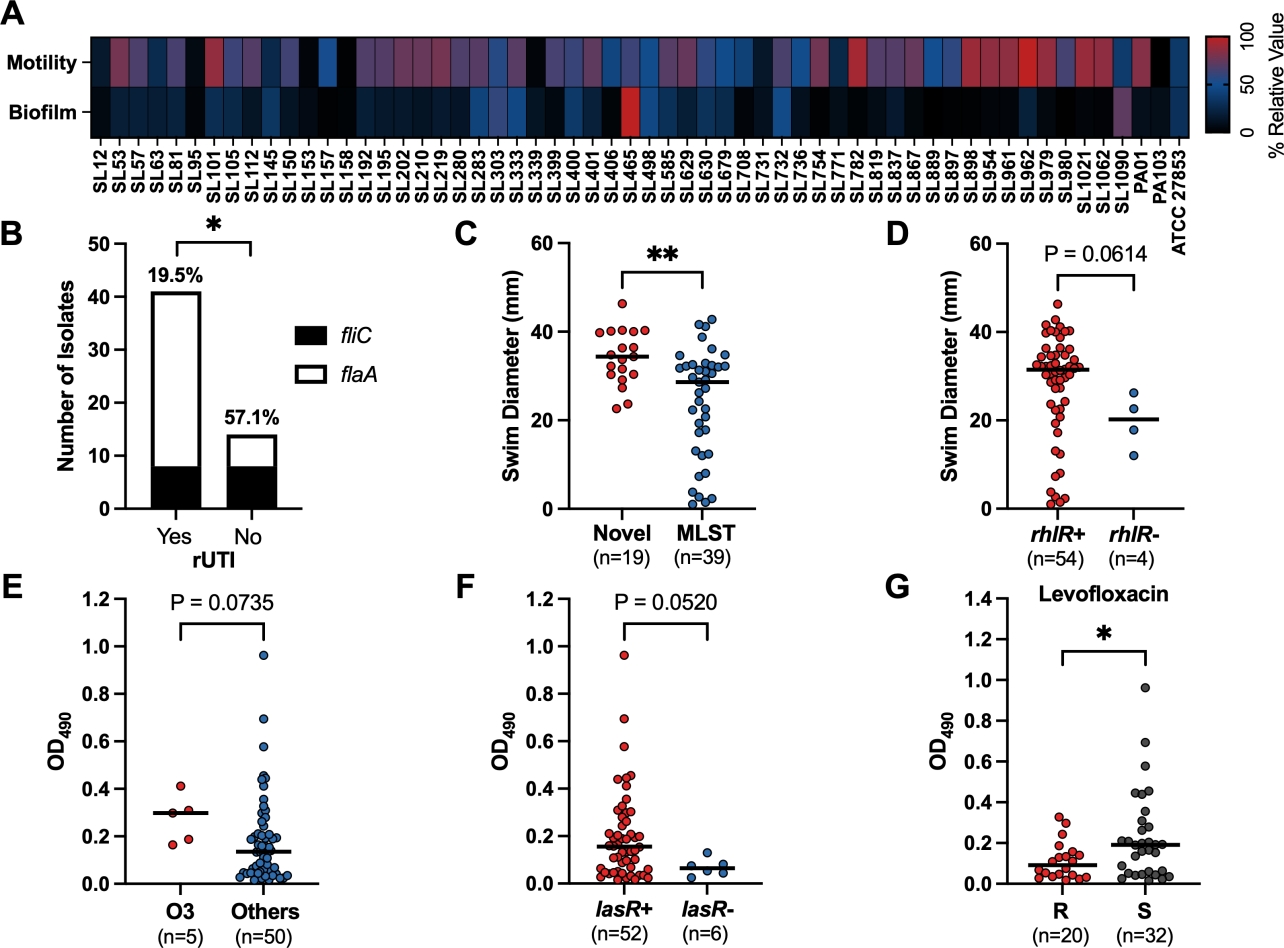
Motility and biofilm phenotypes correlate with virulence genes, levofloxacin resistance, and recurrent UTI. (**A**) Heatmap of normalized motility swim diameter (mm) and biofilm formation in LB (optical density at 490 nm). Data were normalized to the smallest and largest values in each data set. (**B**) Prevalence of type A flagellin (*flaA,* white) and type B flagellin (*fliC*, black) in strains isolated from patients with recurrent UTI (rUTI) (red) compared with patients without rUTI (blue). Motility (swim diameter) in strains with (**C**) novel ST types (red) vs those with established MLST (blue) and (**D**) isolates with a PAO1-like RhlR homolog (red) compared to those without (blue). Dots represent the mean of biological replicates for a strain, and bars indicate median. Biofilm formation (OD_490_) in strains (**E**) of the O3 serotype (red) compared to other serotypes (blue), (**F**) with (red) vs without (blue) BLAST detection of LasR, and (**G**) resistant (R, red) vs susceptible (S, black) to levofloxacin. Statistical significance was determined via Fisher’s exact test for panel **B** and Mann-Whitney *U* test for panels **C–G** (*P < 0.05, **P < 0.01).

Motility and biofilm formation are central to *P. aeruginosa* persistence in the urinary tract, allowing tissue colonization and evasion of antibiotic clearance (1, 9, 45). Despite all isolates encoding the flagellar machinery, five isolates were non-motile, while the rest demonstrated varying levels of motility (Fig. 5A). Isolates from patients with recurrent urinary tract infections (rUTI) were 5.5 times more likely to encode the type A flagellin gene (*flaA*), whereas isolates from non-rUTI patients were more likely to carry type B flagellin (*fliC*) (Fig. 5B; Fig. S6). This association remained significant after multivariable adjustment (OR 5.94, 95% CI 1.57–25.19; Table S1). Novel ST isolates exhibited 20.2% greater swim motility compared to those with established STs (Fig. 5C) and increased motility was associated with the detection of a PAO1-like RhlR homolog (Fig. 5D). Biofilm formation was 2.2 and 2.4 times higher in strains of the O3 serotype (Fig. 5E) and in strains with PAO1-like LasR (Fig. 5F), respectively. In contrast, isolates resistant to levofloxacin exhibited 51.8% weaker biofilm formation than those that were susceptible (Fig. 5G). These trends suggest that strain background shapes motility and biofilm phenotypes that may support persistence in the urinary tract.

### Swimming motility and *exoS* presence correlate with colonization in a murine model of UTI

**Fig 6:**
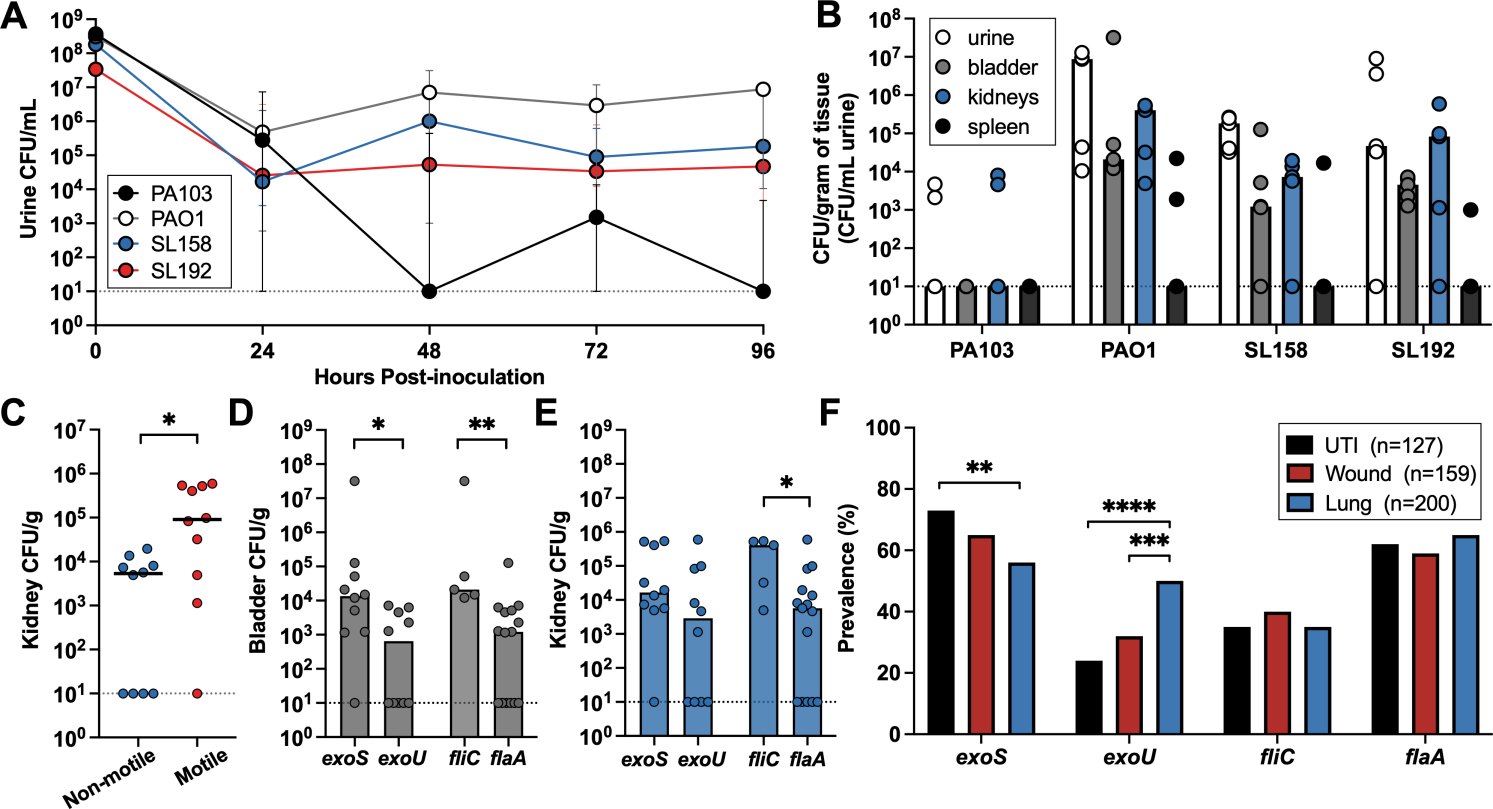
Murine UTI model links virulence traits to bacterial burden. Female CBA/J mice (*n* = 20) were transurethrally inoculated with 2 × 10^8^ CFU of *Pseudomonas*, either PAO1 (*n* = 5), PA103 (*n* = 5), SL158 (*n* = 5), or SL192 (*n* = 5). (**A**) Urine samples were collected from each mouse every 24 h post-infection and plated to determine CFU burden. Mean urine CFU of biological replicates (*n* = 5) is displayed (dot) ± SEM. The limit of detection is indicated by a dashed line. Mice were sacrificed after 96 h, and organs were aseptically collected, homogenized, and plated to determine CFU per gram of tissue. (**B**) 96-h organ CFU and urine CFU/mL for each mouse (dot) are indicated (*n* = 20) with bar height indicating median and a dashed line indicating the limit of detection. (**C**) Plot of kidney CFU burden in non-motile (PA103, SL158) compared to motile (PAO1, SL192) strains. (**D**) Bladder and (**E**) kidney CFU/g stratified by exotoxin genotype (*exoS* vs *exoU*) and flagellin type (*fliC* vs *flaA*). Significance was determined by Mann-Whitney *U* test for panels **C–E** (*P < 0.05, **P < 0.01). (**F**) Bar graph showing the proportion of *exoS*, *exoU*, *fliC*, and *flaA* positive *P. aeruginosa* isolates stratified by infection type: UTI (black), wound (red), and lung (blue). Significance was determined with Fisher’s exact test (**P < 0.01, ***P < 0.001).

To complement our *in vitro* assay findings, we used the traditional ascending murine UTI model to directly assess how phenotypic and genotypic characteristics contribute to colonization and persistence *in vivo* (46). Four *P. aeruginosa* strains (PAO1, PA103, SL158, and SL192) were selected to represent distinct T3SS toxin profiles and transurethrally inoculated into CBA/J mice (*n* = 20) (Fig. S7). PAO1 maintained the highest urine CFU burden, while PA103 declined over 96 h (Fig. 6A; Fig. S8). A similar pattern was seen in tissue CFU burdens, where PA103 had reduced organ colonization, clinical isolates displayed intermediate levels, and PAO1 had the highest burdens (Fig. 6B). Kidney colonization differed by motility phenotype (Fig. 6C), with motile strains colonizing the kidney at median levels over one log higher than non-motile strains. Strains lacking the toxin gene *exoS* exhibited a 100-fold decrease in the urine (Fig. S9A) and a 5-fold decrease in both bladder (Fig. 6D) and kidney (Fig. 6E) colonization compared to *exoS*-positive strains. Additionally, isolates encoding type B flagellin (*fliC*) showed a 10.9-fold advantage in bladder colonization (Fig. 6D) and a 13.5-fold advantage in kidney colonization (Fig. 6E) relative to those encoding type A flagellin (*flaA*). In contrast, bacterial burdens in the spleen were low and did not differ between exotoxin or flagellin groups (Fig. S9B). To contextualize these *in vivo* findings, we compared the prevalence of these same exotoxin and flagellin genes between our cohort and publicly available U.S. urinary isolates as well as across *P. aeruginosa* genomes from other infection sources from the BV-BRC database. Our cohort exhibited a higher prevalence of *exoS* than the broader U.S. urinary strain collection (Fig. S10), and global UTI isolates were likewise significantly enriched for *exoS* compared to lung isolates (Fig. 6F). Collectively, these findings indicate motility and exotoxin ExoS may be important contributors to *P. aeruginosa* ascension, colonization, and persistence in the uncomplicated urinary tract model.

## DISCUSSION

In this study, we performed extensive genotypic and phenotypic characterization of *Pseudomonas aeruginosa* strains isolated from urinary tract infections (UTIs) within our regional healthcare system. While *P. aeruginosa* has been widely studied in the context of respiratory and wound infections, its role in UTIs has been understudied by comparison. The absence of a dedicated UTI reference strain underscores this gap. Indeed, our experimental work relied on type strains derived from wound (PAO1), lung (PA103), and bloodstream (PA27853) infections (30–33), demonstrating the need for dedicated UTI reference strains and models to advance understanding of *P. aeruginosa* uropathogenesis.

Interestingly, 34.5% of isolates in our cohort represented novel sequence types (ST), indicating previously uncharacterized pathogen lineages potentially circulating in our region. This is in comparison to a recent study that examined 528 *P. aeruginosa* genomes and found 20/249 (8.7%) STs to be novel (47). Despite displaying shared phenotypic traits, phylogenetic analysis revealed these isolates did not cluster into a single clade, indicating they do not represent a distinct regional lineage. This observation suggests that the higher prevalence of *P. aeruginosa* UTIs in our healthcare system may reflect host, healthcare, or environmental factors that facilitate urinary tract colonization.

Beyond genomic diversity, we also observed variation in surface antigen types across the clinical isolates. O-antigen serotyping identified eight distinct serotypes in our cohort, with O6, O11, and O5 comprising the majority of isolates. These serotypes have also been among the most prevalent in ventilator-associated pneumonia and burn wound isolates (26, 27, 48). We found that O6 strains were more common in patients with indwelling urinary catheters (IDCs) and were independently linked to hematuria. Notably, the O6 antigen contains an amide group and requires ammonia for biosynthesis, derived either from environmental sources or glutamine catabolism (49), suggesting a potential link to nitrogen availability in the urinary environment. Given the modest cohort size, these findings should be considered exploratory and require validation in larger studies.

Antibiotic resistance patterns in our patient population were consistent with those in healthcare-associated infections (50), but we identified novel associations with host demographics. In our cohort, isolates from African American patients exhibited significantly higher resistance to meropenem and aztreonam than those from White patients. While the literature on racial and ethnic disparities in antibiotic resistant infections is limited (51), recent studies of resistant pathogens have suggested that demographic patterns in antimicrobial resistance may relate to broader social and clinical exposures rather than race alone (52, 53). The pattern observed here may, therefore, reflect differences in prior exposures, healthcare-associated risk, or other epidemiological factors that warrant further investigation. In addition, strains of serotype O11 were more likely to be extensively drug-resistant (XDR), consistent with previous studies correlating O11 to antibiotic resistance (54). As described in other infection models (55, 56), we observed a trade-off between antibiotic resistance and biofilm formation. Resistance to levofloxacin, aztreonam, or meropenem was associated with reduced hemolytic activity, suggesting additional possible fitness trade-offs between resistance and cytotoxicity.

To expand the AMR analyses beyond the intrinsic resistance detected by CARD, we screened isolates for common SNP mutations associated with fluoroquinolone resistance. Mutations in the examined loci only explained a subset of fluoroquinolone resistance in our cohort, consistent with prior studies (57). Among the variants identified, GyrA T83I was the most common, accounting for ~47% of levofloxacin resistance (39, 40). The remaining resistance likely reflects additional chromosomal mechanisms not assessed here, including mutations outside QRDR loci and efflux-related changes (5, 58). Nevertheless, our genomic and phenotypic data provide a valuable resource for future studies to examine genomic islands or mobile genetic elements aimed at resolving co-acquired virulence and resistance in urinary *P. aeruginosa* isolates.

To date, there is a paucity of literature utilizing *P. aeruginosa* in UTI models, with most prior work focused on catheter-associated infection (59, 60). In CAUTI models, urine represses quorum sensing, and mutants can colonize as efficiently as wild-type strains (61). In contrast, studies in LACA mice have shown quorum sensing systems including PQS, Rhl, and Las are crucial for *P. aeruginosa* virulence during acute infection (16, 62). Importantly, *lasR* loss-of-function (LOF) mutations that impair quorum sensing and protease expression are a hallmark of chronic *P. aeruginosa* infections such as cystic fibrosis (63, 64), and PA103 carries such a *lasR* mutation (65). To determine which phenotypic traits influence urinary tract colonization *in vivo*, we utilized the CBA/J murine model of ascending UTI.

In the CBA/J model, previously only used in the context of *P. aeruginosa* co-infections (66, 67), swimming motility correlated with urinary tract colonization. This pattern aligns with observations in other uropathogenic species (3, 68) and is consistent with the established role of motility in *P. aeruginosa* dissemination across multiple infection sites, including lung (45, 69), wound (70), and burn (71) models. Interestingly, flagellin type also correlated with recurrent UTI; *flaA* was enriched in isolates from patients with recurrent UTIs, whereas *fliC* was more common in acute cases. This may indicate selection to avoid immune recognition of FliC by TLR5 (72). Indeed, FliC is very immunogenic and has proven effective as a therapeutic agent against *P. aeruginosa* in experimental models (73–76). Larger *in vivo* cohorts will be necessary for refining these conclusions and clarifying the mechanisms of *P. aeruginosa* dissemination and persistence in the urinary tract, as has been done in similar studies with *E. coli* (77).

In addition to motility, we observed differences in colonization associated with exotoxin profiles. In a previous study, the type III secretion system (T3SS), specifically ExoU injection, was found to be the main contributor to virulence in acute catheter-associated urinary tract infection (CAUTI) (61). The T3SS is also important in lung and wound pathogenesis, but there is debate over whether *exoS* or *exoU* plays a larger role (78–80). In our murine UTI model, *exoS*-positive strains (SL158, PAO1) exhibited higher bacterial burdens than *exoU*-positive strains (SL192, PA103), suggesting that encoding *exoS* may be advantageous for urinary tract colonization. Notably, we also observed a higher prevalence of *exoS*-positive strains in our regional data set compared to publicly available U.S. urinary isolates. This likely contributed to the higher prevalence of *exoS* among urinary isolates compared to lung isolates in our comparative genome analysis. Future work will determine the mechanistic and functional outcomes of ExoS activity in the UTI model.

While previous studies have evaluated PAO1 in the murine UTI model (16, 17), to our knowledge, none have assessed PA103. Despite exhibiting high virulence in pneumonia models (81, 82), PA103 was the only strain unable to colonize the murine urinary tract, posing the question of what strain characteristics are contributing to this defect. PA103 is non-motile due to a single amino acid substitution in FleQ (83), a mutation absent from the other three strains tested *in vivo*. In fact, none of our clinical urinary strains exhibited this mutation, further supporting the importance of motility to *P. aeruginosa* urovirulence. Because PA103 is also *exoS*-negative, it remains difficult to disentangle the relative contributions to its colonization defect. Future murine studies restoring motility in PA103 via *fleQ* repair and providing *exoS* in *trans* will clarify the relative contributions of motility and exotoxin function in colonization.

Overall, our data reveal that *P. aeruginosa* UTIs in our region are caused by a genetically diverse set of isolates, including novel lineages with distinct combinations of virulence and resistance traits. By integrating genomic, phenotypic, and *in vivo* analyses, we provide new insights into how *P. aeruginosa* adapts to and colonizes the urinary tract. These findings support recognition of *P. aeruginosa* as a clinically relevant uropathogen and highlight the need for continued investigation into this underexplored role.

## MATERIALS AND METHODS

### Bacterial strains, media, and culture conditions

Bacterial strains used in this study included clinical isolates of *Pseudomonas aeruginosa* collected under University of South Alabama (USA) IRB protocol #2178590. All clinical isolates were obtained from the University Hospital from excess clinical urine specimens collected as part of routine care. Species identification was confirmed via oxidase testing and/or matrix-assisted laser desorption/ionization time-of-flight (MALDI-TOF). Isolates were de-identified, date-blinded, coded, and stored at -80°C in Luria broth (LB) containing 20% glycerol. Strains were cultured from a single colony in LB at 37°C with aeration at 200 rpm. In experiments requiring urine, filter-sterilized (0.22 µm) pooled human urine from at least six healthy de-identified female donors was used (deemed non-human subjects research by USA).

### Antibiotic susceptibility testing, urinalysis, and patient metadata

Antimicrobial susceptibility testing (AST) and urinalysis were performed at the University of South Alabama hospital microbiology laboratory. AST was performed using automated broth dilution (BD Phoenix M50) following Clinical and Laboratory Standards Institute (CLSI) guidelines (84). Strains were categorized as resistant (R), intermediate (I), or susceptible (S) based on minimum inhibitory concentrations (MICs) (Table S1). Multidrug resistant (MDR) was defined as resistance to ≥1 agent in ≥3 antimicrobial classes (6), and extensively drug-resistant (XDR) was defined as resistance to all but one or two antimicrobial classes in the AST panel. Urinalysis was performed via dipstick chemical analysis and microscopy.

Urine culture, AST, and urinalysis results associated with clinical isolates were obtained via retrospective chart review. Causative agents, antibiotic susceptibilities, and dipstick results were recorded. De-identified patient variables, including patient age, race, sex, and body mass index (BMI), were extracted from the medical record by an honest broker and linked to the corresponding coded isolate (IRB protocol #2178590).

### Biofilm assay

Biofilm formation was assessed using a crystal violet staining assay. Overnight cultures were diluted 1:100 into LB in duplicate in 12-well plates. Plates were sealed with a sterile gas-permeable membrane and incubated statically at 37°C for 24 h. Wells were washed with 1× phosphate-buffered saline (PBS), stained with 0.1% crystal violet for 10 min, and washed again. Biofilm biomass was quantified by solubilizing the dye with ethanol and measuring absorbance via optical density (OD_490_) using an Agilent BioTek 800 TS Absorbance Reader.

### Iron acquisition, hemolysis, and motility assays

Iron chelation was measured using the Chrome Azurol S (CAS) assay. Overnight cultures (5 µL) were spotted onto CAS plates (85) and incubated at 37°C for 16 h, and halo diameters were measured (mm). Hemolytic activity was evaluated by spotting 5 µL overnight bacterial cultures onto 5% sheep blood agar plates (Hardy Diagnostics CAT# A10) and measuring zones of clearance (mm) after 16-h incubation at 37°C. Motility assays were performed in semi-solid tryptone agar (10 g tryptone, 5 g sodium chloride, 2.5 g agar per liter). Cultures were normalized to OD_600_ = 1.0 in HEPES buffer (pH 8.4), stabbed into agar, and incubated at 30°C. Swim diameter was measured (mm) after 16 h.

### Growth curve assay

Overnight cultures were washed in 1× PBS and diluted 1:100 into LB or filter-sterilized, pooled human urine in triplicate in 96-well plates. Plates were sealed with sterile gas-permeable membranes, and growth was monitored at OD_600_ every 10 min for 24 h using a BioTek LogPhase600 Microbiology Reader. Summary statistics for all phenotypic assays are provided in Data set S4.

### Murine model of UTI

Female CBA/J mice (6–8 weeks, Jackson Laboratories) were anesthetized with ketamine/xylazine and transurethrally inoculated with 50 µL of 2 × 10^8^ CFU/mL suspensions of strain PAO1, PA103, SL158, or SL192 with a sterile polyethylene catheter connected to an infusion pump (46, 86). Urine samples were collected and plated on LB agar every 24 h post-inoculation to assess bacterial load. After 96 h, mice were euthanized, and bladders, kidneys, and spleens were aseptically harvested, homogenized, serially diluted, and plated to quantify bacterial burden. All protocols were approved by the Institutional Animal Care and Use Committee (IACUC #2006187) at the University of South Alabama.

### Whole-genome sequencing

Genomic DNA was extracted using the Promega Wizard Genomic DNA Purification Kit following the manufacturer’s instructions. Libraries for isolates SL12-SL708, SL732, and SL736 were prepared using the Nextera XT DNA Library Preparation Kit and sequenced by Moderna TX via the Illumina Nextera XT system to produce 2 × 150 bp paired-end reads. Isolates SL731 and SL754-SL1090 were sequenced by SeqCoast Genomics, where DNA libraries were prepared using the Illumina DNA Prep tagmentation kit with Illumina Unique Dual Indexes according to the manufacturer’s protocol. Libraries were sequenced on the Illumina NextSeq 2000 platform using a 300-cycle flow cell kit to produce 2 ×150 bp paired-end reads.

### Genome assembly and annotation

Sequencing reads were processed using FastQC (87), and genome assemblies were generated with Unicycler (88) v0.4.8 on BV-BRC (89) v3.57.10 using an annotated recipe for the *P. aeruginosa* taxonomy. Eight assemblies were flagged as poor quality on BV-BRC after this process and were reprocessed using an additional polishing workflow. Reads were trimmed with fastp (90) v0.23.4 and taxonomically filtered with Kraken2 (91) v2.1.3 to retain *Pseudomonas* (taxid 286). Genomes were assembled using SPAdes (92) v3.15.5 and polished once with Pilon (93) v1.24 using bwa-mem2 read-mapping. Contigs shorter than 1 kb were removed, and assembly metrics were assessed with QUAST (94) v5.2.0. Following this polishing process, all assemblies except one (SL192) improved in quality and were no longer flagged by BV-BRC. SL192 remained more fragmented but was retained because it was included in phenotypic assays and murine infection experiments. Pre- and post-polishing metrics for the eight assemblies flagged during initial processing are summarized in Data set S5, and metrics for all 55 final assemblies are summarized in Data set S6. All assemblies were subsequently annotated using the BV-BRC RAST tool kit (RASTtk) (95).

### Bioinformatics analysis

Virulence genes were identified by BLASTP against reference proteins from *Pseudomonas aeruginosa* PAO1 (Table S4). Matches with ≥85% amino acid identity and ≥90% query coverage were considered homologous sequences (hits). For SL192, loci were manually inspected to confirm that the expected sequences were present and not truncated across contig boundaries. Because T3SS effector genes vary across *P. aeruginosa* lineages (96), multiple reference strains (PAO1, PA103, and ATCC 27853) were used for BLAST detection (Table S4). Antimicrobial resistance genes were identified with the Comprehensive Antibiotic Resistance Database (CARD) Resistance Gene Identifier (RGI) (37) v6.0.5. “Perfect” matches represent exact matches to curated resistance genes, while “Strict” matches represent high-confidence matches exceeding the detection threshold defined by CARD models. For the *parS* locus used in genotype-phenotype analyses, the presence was determined using BLAST similarity to the PAO1 reference sequence due to inconsistent automated detection by CARD.

Whole-genome SNPs were identified using kSNP4 (BV-BRC). The optimal k-mer size was selected automatically by the software (*k* = 19). SNPs were retained if present in ≥80% of genomes. A maximum-likelihood phylogeny was inferred from the resulting SNP matrix, and iTOL (97) v7.5.0 was utilized for editing the tree. ST typing was performed using the PubMLST *Pseudomonas aeruginosa* online typing database (98). Allelic matches are provided in Data set S1. Serotypes were determined using the PAst v1 *in silico* serotyping tool (99). To determine the presence of genetic mutations, multiple sequence alignment was performed via Mafft (100) v7.526.

Two independent data sets of publicly available *P. aeruginosa* genomes were retrieved from BV-BRC (PATRIC). For virulence gene comparisons across infection sites (Fig. 6F), genomes were restricted to isolates explicitly annotated as originating from UTI, respiratory, or wound infection sources. For Gulf Coast vs U.S. urinary comparisons (Fig. S2 and S10), all genomes annotated with a urine isolation source were included regardless of whether infection status was specified. Accession numbers and metadata for these groups are provided in Data set S7 and S8.

### Statistical analysis

Statistical analyses were performed using GraphPad Prism (v10.4). Variables were screened for correlation via Pearson’s correlation. For group comparisons, normality was assessed independently within each comparison group using Shapiro-Wilk and D’Agostino-Pearson normality tests. Approximately normally distributed data sets were analyzed using unpaired two-tailed *t*-tests, while non-normally distributed data sets were analyzed using Mann-Whitney U tests (95% CI). Welch-corrected *P* values are reported in figure legends with unpaired *t*-tests for transparency. When comparing categorical variables, Fisher’s exact test was used. *P* values < 0.05 were considered statistically significant. Regression analyses were performed for selected outcomes using logistic or linear regression, as appropriate. For human urine growth, variables significant in univariable analysis were included in the multivariable model. Final model outputs are provided in Tables S3 and S4.

## Data Availability

Raw sequencing data for all isolates haves been deposited in the SRA (PRJNA1390275).

## SUPPLEMENTAL MATERIAL

Supplemental Figures msphere.00151-26-s0001.pdf Figures S1–S10.

Supplemental Data Sets msphere.00151-26-s0002.xlsx Data sets S1–S8.

Supplemental Tables msphere.00151-26-s0003.xlsx Tables S1–S4.

ASM does not own the copyrights to Supplemental Material that may be linked to, or accessed through, an article. The authors have granted ASM a non-exclusive, world-wide license to publish the Supplemental Material files. Please contact the corresponding author directly for reuse.
