# Supplementary material for: Emerging pathogens in urinary tract infections: virulence and phenotypic characterization of Pseudomonas aeruginosa strains
Source: mSphere. 2026 May 13;11(6):e00151-26. doi: 10.1128/msphere.00151-26 (PMC13317196; doi:10.1128/msphere.00151-26)
Supplement: Supplemental Figures — Figures S1–S10. [file msphere.00151-26-s0001.pdf]

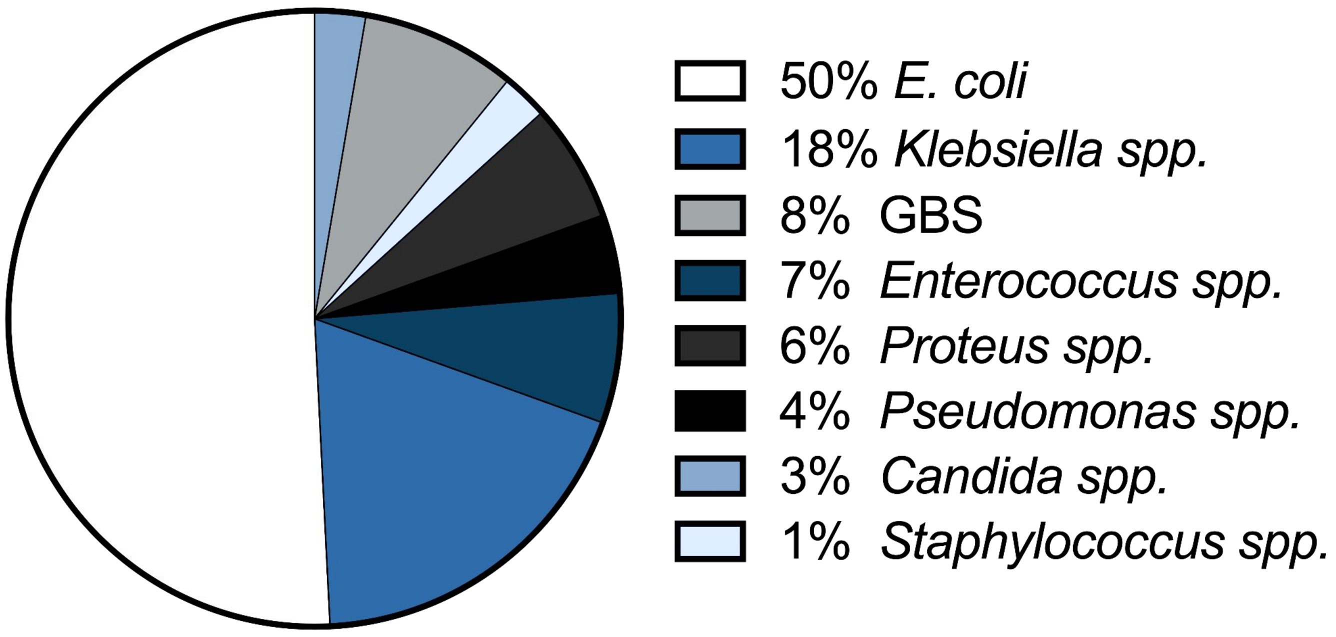

Figure S1. UTI pathogen distribution in the University of South Alabama healthcare system (Mobile, Alabama). Retrospective chart review was conducted to identify the top UTI causative agents across the USA healthcare system from June 1, 2024 to May 31, 2025. Positive UTI cases were identified via urine culture (n=6929). Organisms <1% are not shown.

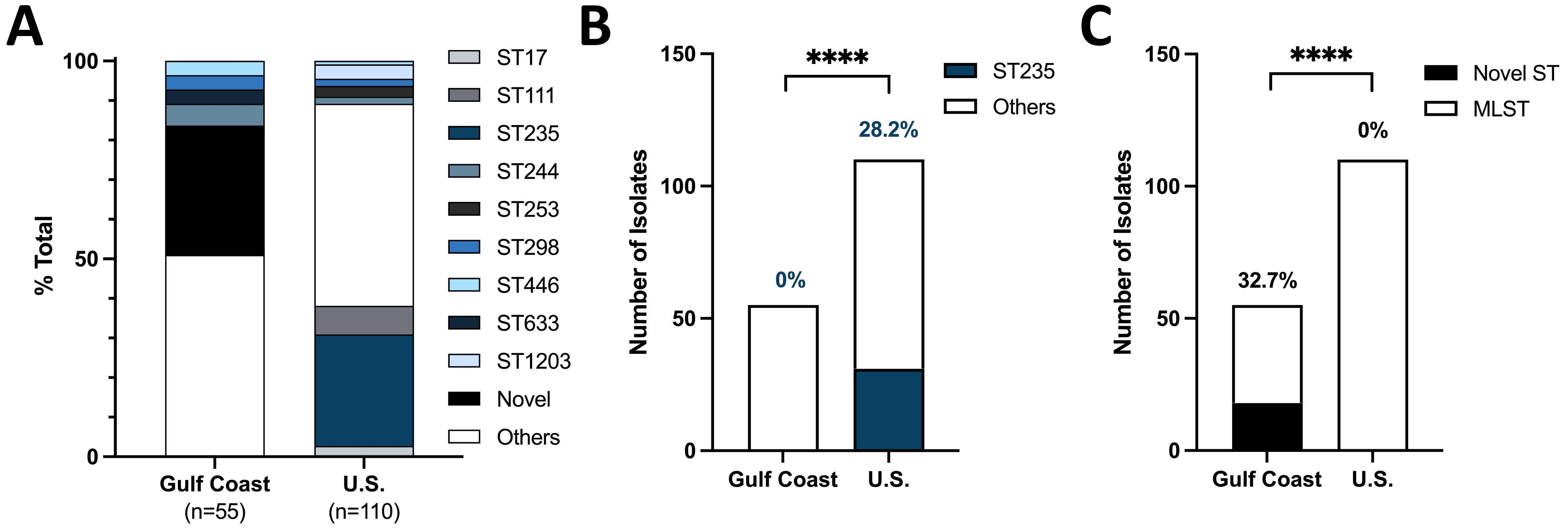

Figure S2. Distribution of sequence types in Gulf Coast urinary isolates compared with U.S. urinary isolates. **(A)** Distribution of top multilocus sequence types (STs) among *P. aeruginosa* urinary isolates from the Gulf Coast cohort compared with urinary isolates from across the United States obtained from the BV-BRC database. ST types comprising <2% of both groups are included as "Others". **(B)** Prevalence of ST235 versus all other STs in Gulf Coast isolates compared with U.S. urinary isolates. **(C)** Proportion of isolates with novel STs in the Gulf Coast cohort compared with isolates with established MLST designations in the U.S. dataset. Percent values above bars indicate the proportion within each group. Statistical significance was determined using Fisher's exact test (****P<0.0001).

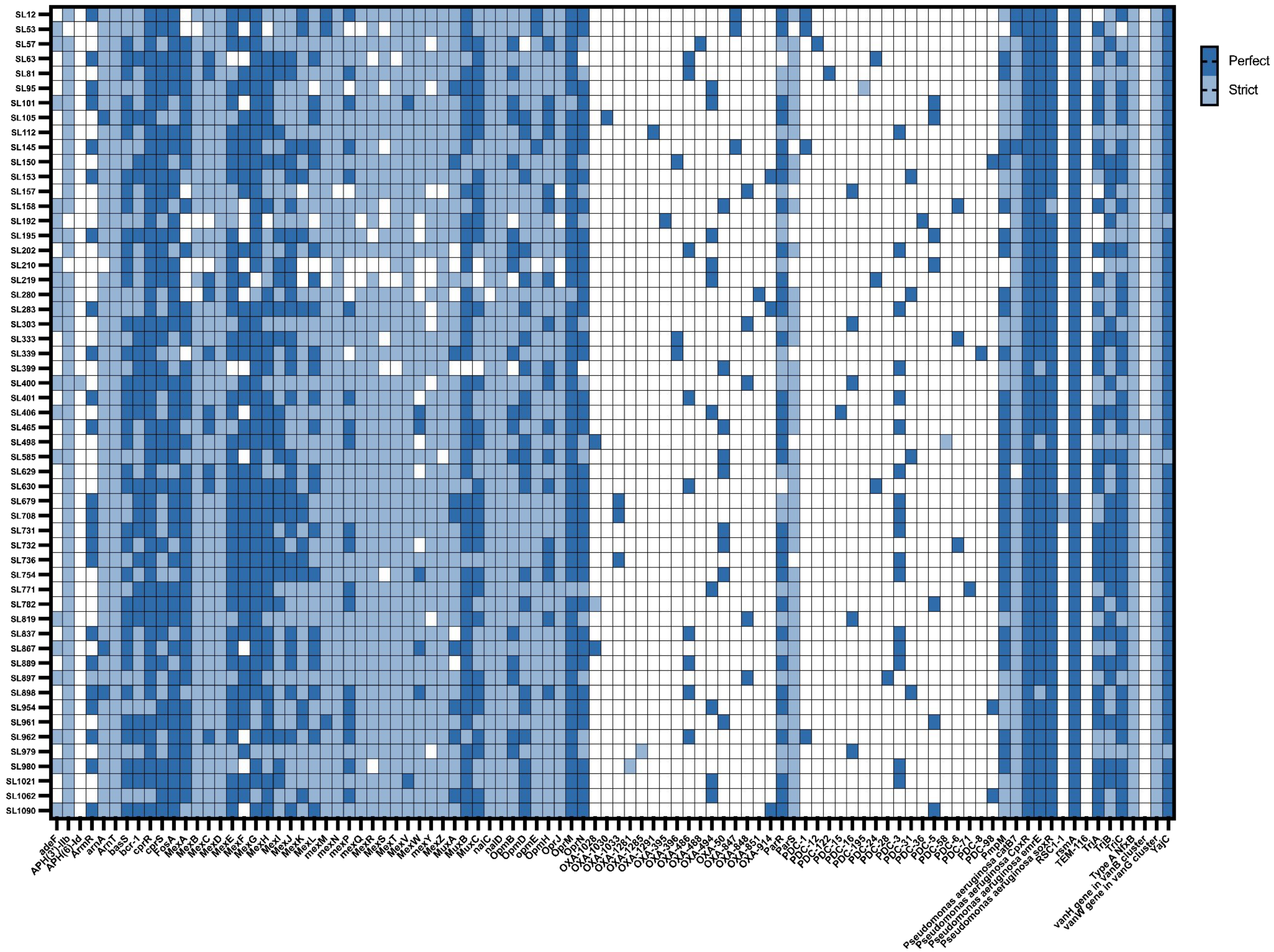

Figure S3. Clinical *P. aeruginosa* strains show high number of antibiotic resistance genes. The genomes of the 55 clinical *P. aeruginosa* isolates were blasted for antibiotic resistance genes in the Comprehensive Antibiotic Resistance Database (CARD). Perfect hits (dark blue) have all amino acids matching the CARD sequence, while strict hits (light blue) indicate very close matches with slight variations in the sequence. White indicates the gene is absent.

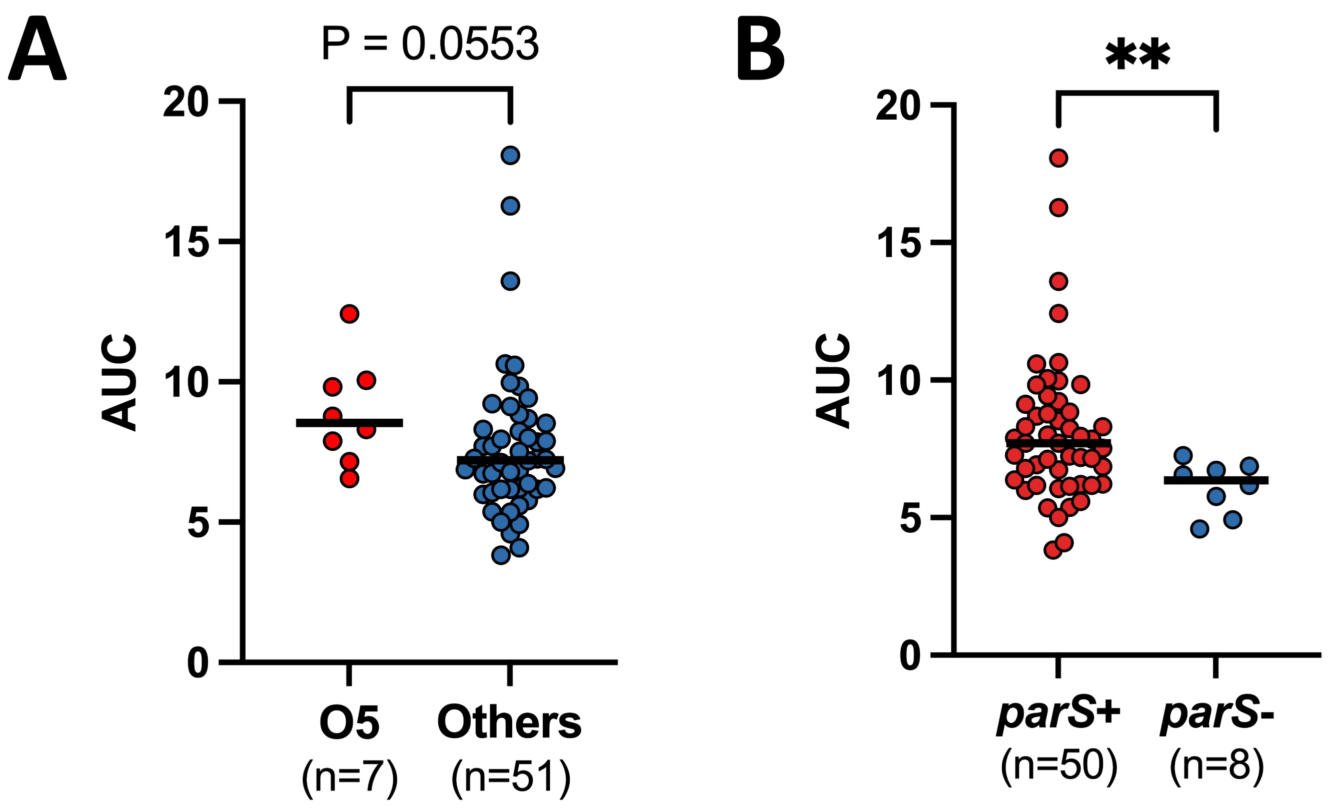

Figure S4. Growth in human urine correlates with O-antigen serotype and ParS detection. (A) Plot of growth in human urine (AUC) in serotype O5 (red) versus all other serotypes (blue). **(B)** Plot of growth in human urine in strains with (red) and without (blue) a BLAST match to ParS. Each dot represents a clinical isolate, and the median of each group is indicated with a black line. Statistical significance was determined via Mann-Whitney U test (**P<0.01).

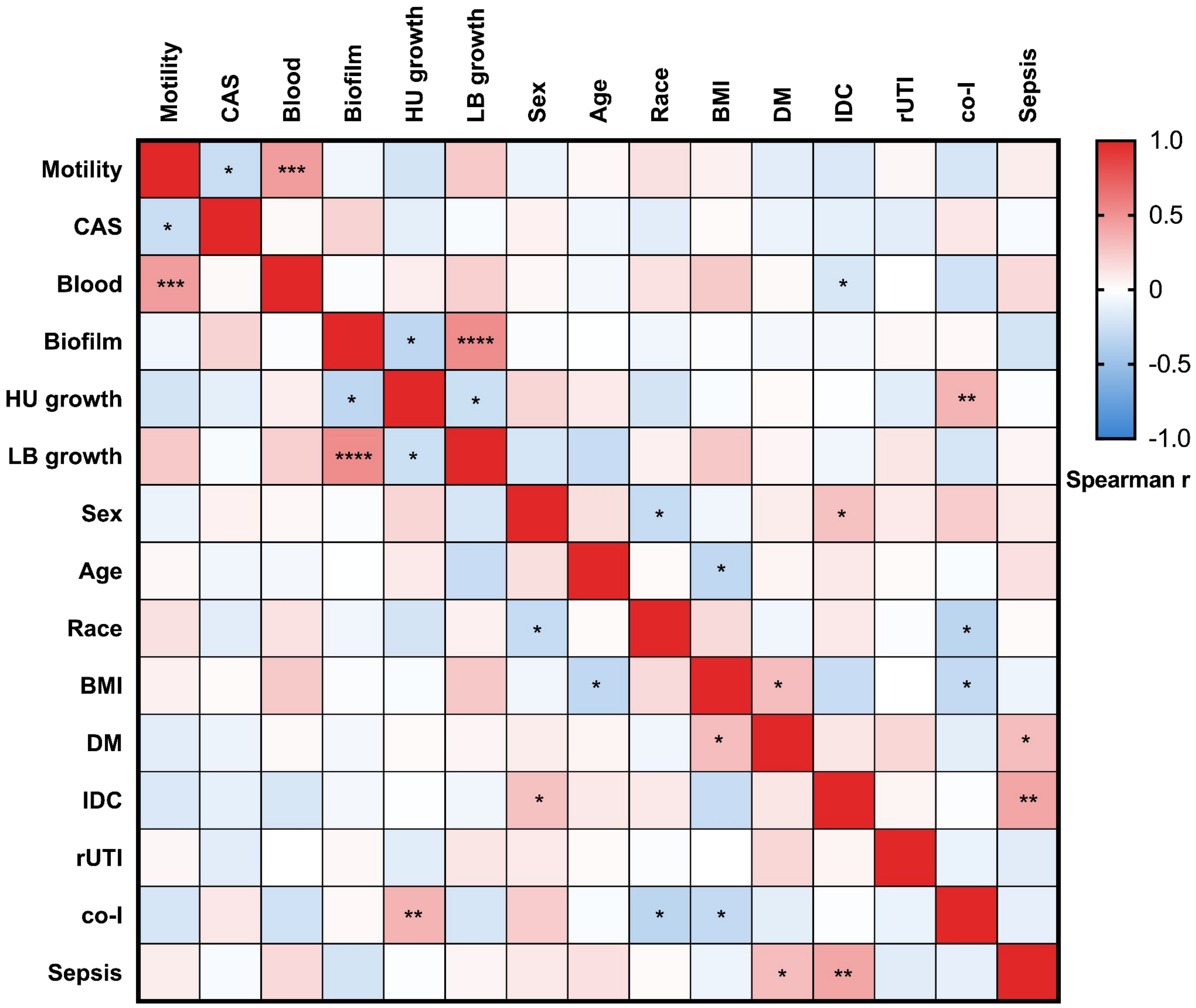

Figure S5. Correlation matrix of phenotypic data to patient variables. The 55 *P. aeruginosa* clinical isolates were subjected to Chrome Azurol S (CAS), blood agar, biofilm, and growth curve assays to assess phenotype. Patient variables were obtained and tested for correlation with phenotypic data with Spearman r. Red indicates a positive correlation, and blue indicates a negative correlation. P values were determined with 95% confidence (*P<0.05, **P<0.01, ***P<0.001).

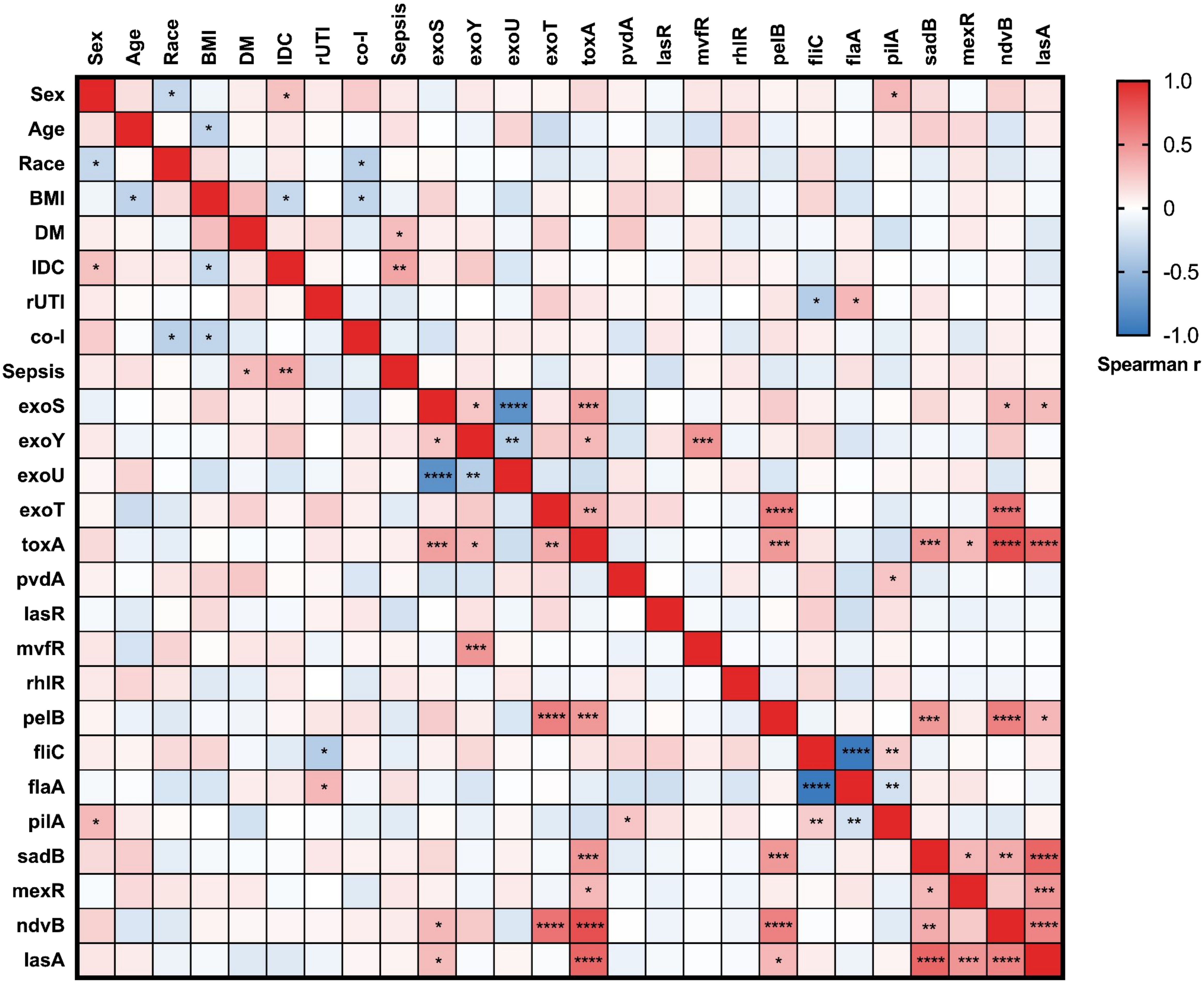

Figure S6. Correlation matrix of genotypic features to patient variables. Genotypic variables were derived from BLASTP analyses. Protein sequences meeting the predefined identity (>85%) and coverage (>90%) thresholds were used to infer corresponding gene presence or absence as a binary variable. These variables were compared with patient variables using Spearman correlation (r). Red indicates a positive correlation, and blue indicates a negative correlation. P values were determined with 95% confidence (*P<0.05, **P<0.01, ***P<0.001, ****P<0.0001). Blank or excluded comparisons are indicated with an X through the cell.

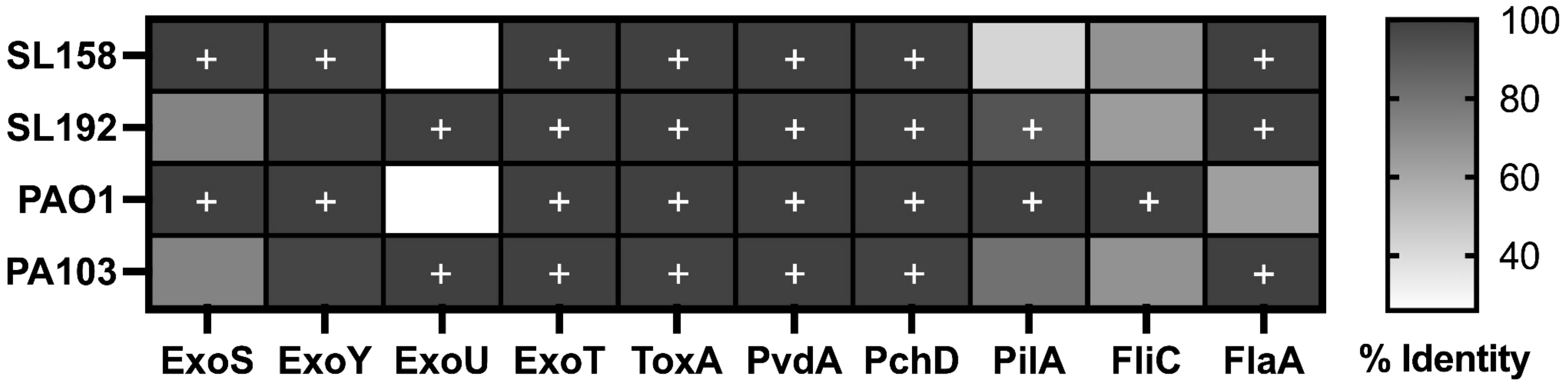

Figure S7. Amino acid identity of virulence-associated gene products in strains used in the murine UTI model. Heatmap showing percent amino acid identity of selected virulence-associated proteins in strains SL158, SL192, PAO1, and PA103 relative to the reference sequences used for BLASTP analysis (Table S2). Amino acid identity is represented on a color scale from low (white) to high (black). Hits above the identity and query coverage thresholds (85% and 90%, respectively) are indicated by “+”.

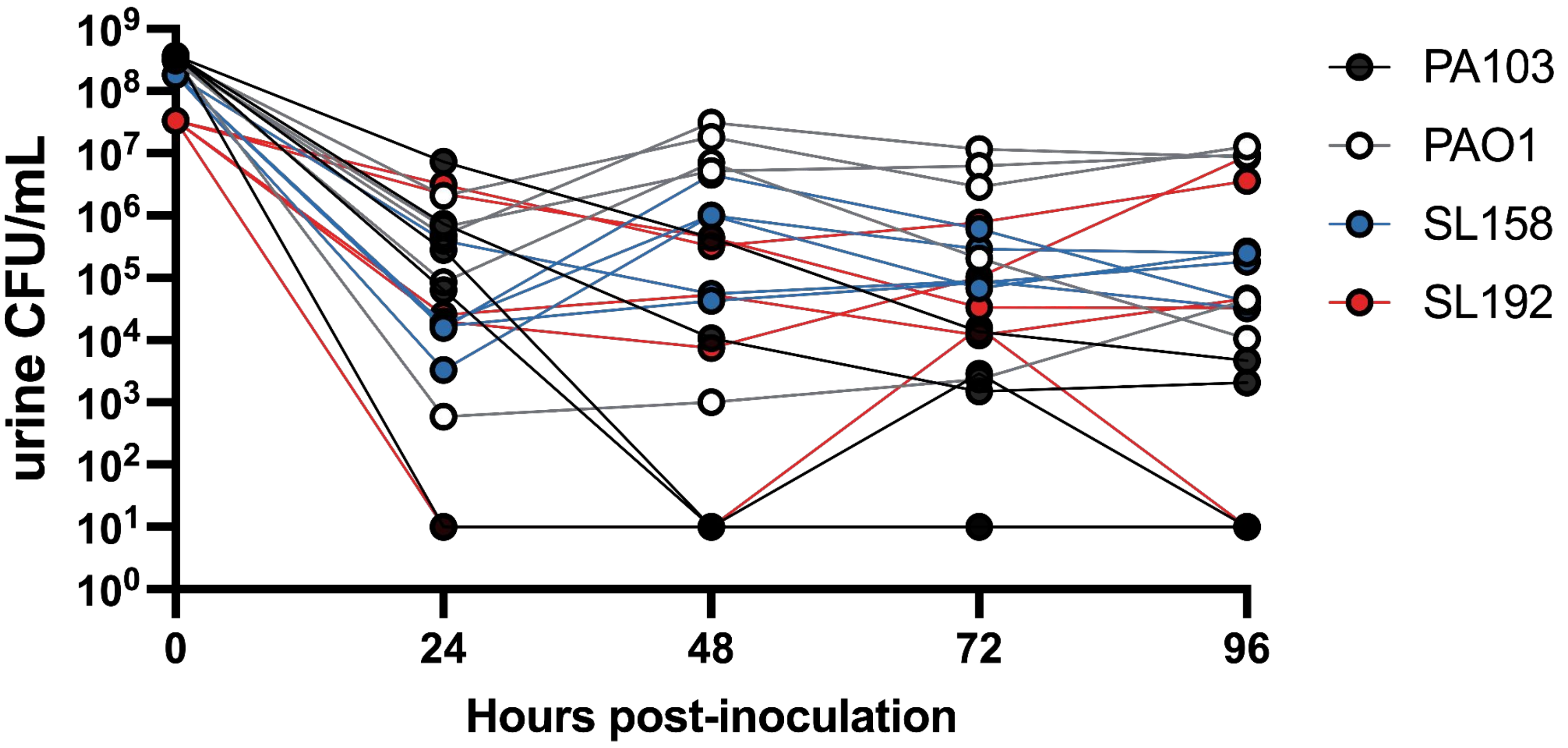

Figure S8. Murine urine CFU burden of *P. aeruginosa* clinical and type strains. Urine samples were collected and plated every 24 hours post-inoculation to quantify bacterial load. Colors indicate strain identity (PA103, black; PAO1, white; SL158, blue; SL192, red). Each symbol represents the mean of three technical replicates for each individual mouse (n=5 mice per strain).

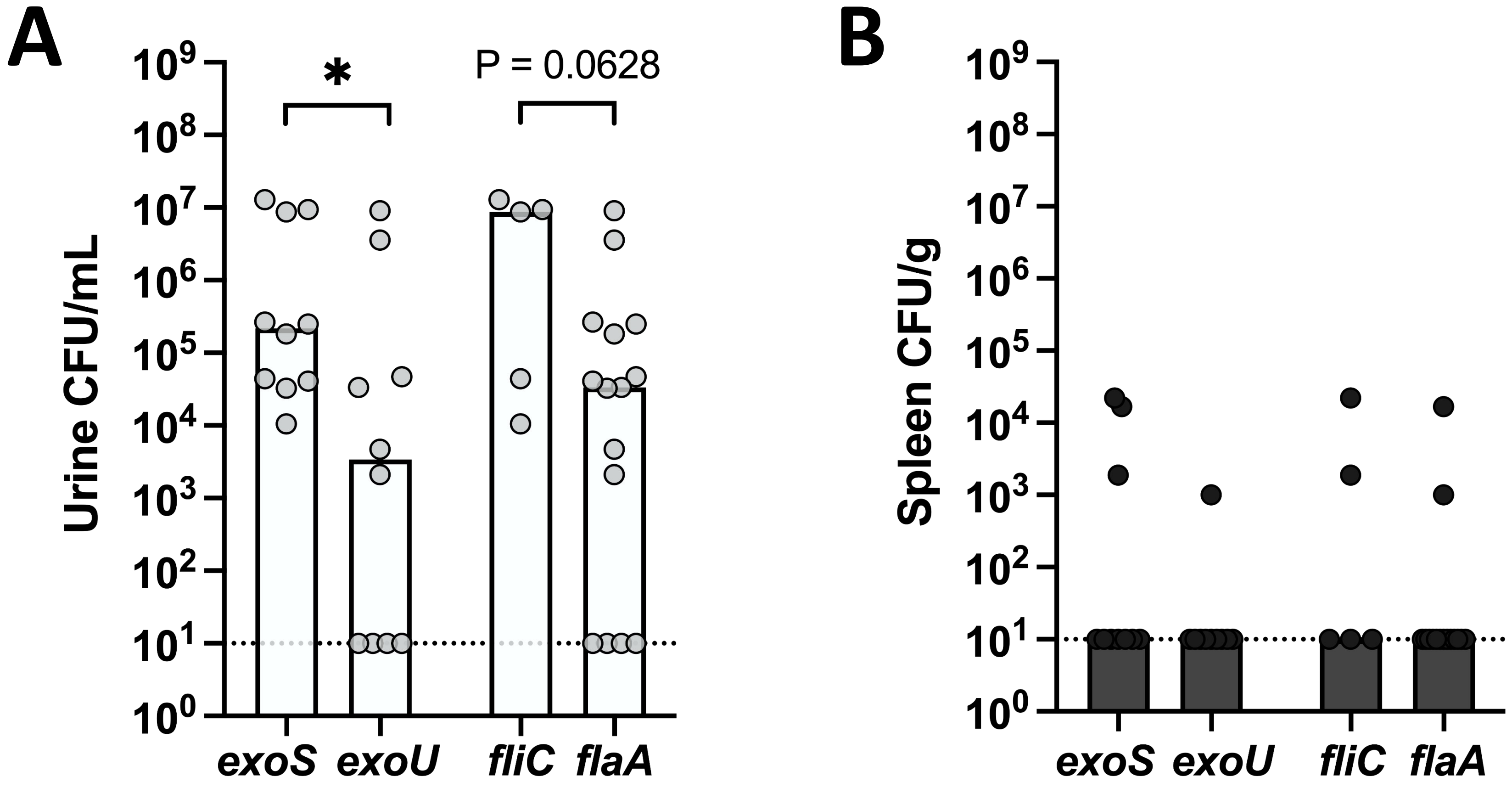

Figure S9. Urine and spleen bacterial burden stratified by exotoxin and flagellin genotype. (A) Urine CFU/mL in mice infected with strains encoding *exoS* (n=10) versus *exoU* (n=10) and type B flagellin (*fliC*) (n=5) versus type A flagellin (*flaA*) (n=15). **(B)** Spleen CFU/g in the same mice following infection. Each dot represents an individual mouse. Bars indicate median values, and the dashed line indicates the limit of detection. Statistical significance was determined using the Mann-Whitney U test (*P<0.05).

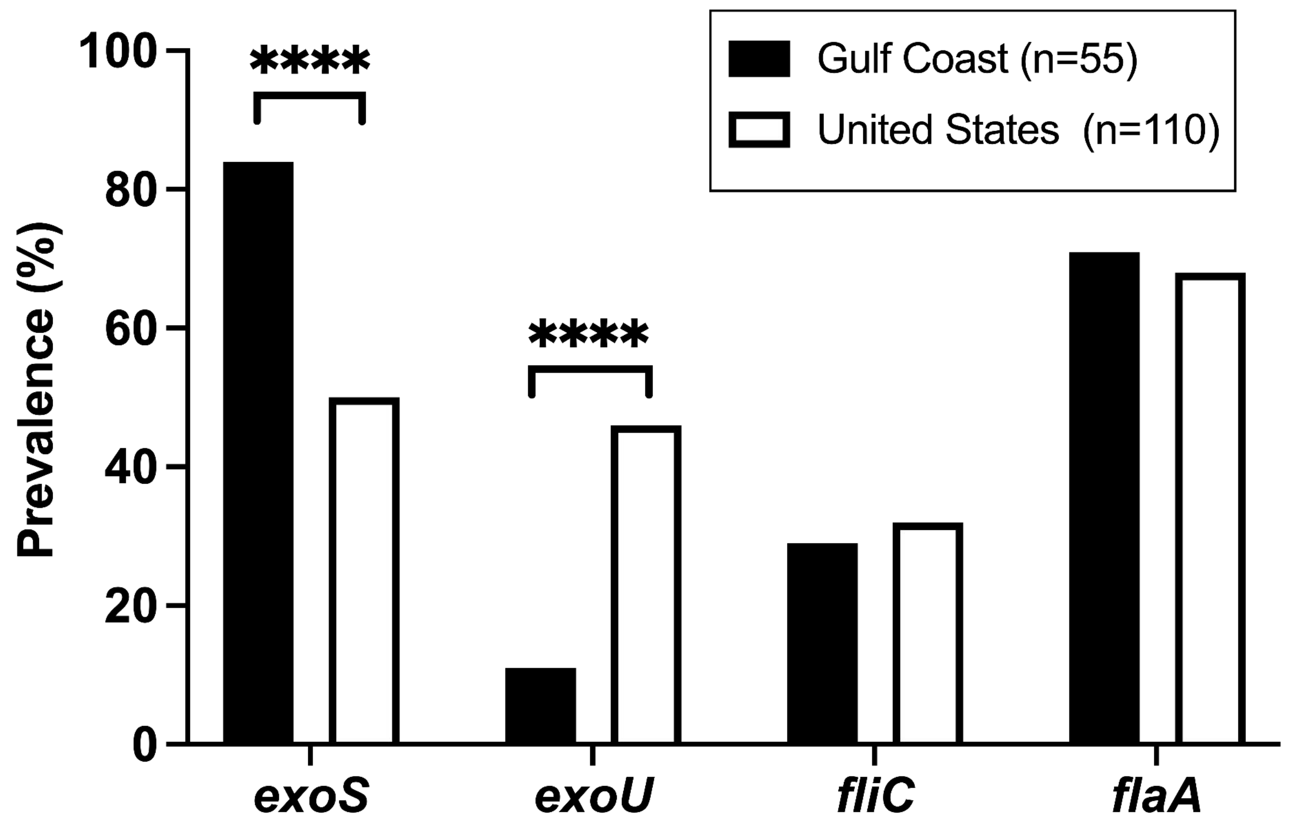

Figure S10. Prevalence of exotoxin and flagellin genes in Gulf Coast urinary isolates compared with U.S. urinary isolates. Prevalence of *exoS*, *exoU*, *fliC*, and *flaA* in our Gulf Coast cohort (black) compared with urinary *P. aeruginosa* isolates from the United States obtained from the BV-BRC database (white). Gene classification was determined based on BLAST matches meeting the predefined amino acid identity and query coverage thresholds. Bars indicate the percentage of isolates within each dataset meeting these criteria. Statistical significance was determined using Fisher's exact test (***P<0.001, ****P<0.0001).
